# *SGS1-SuOff* rescues the mild methylmethane sulfonate sensitivity of *srs2*Δ** cells in *Saccharomyces cerevisiae*

**DOI:** 10.17912/micropub.biology.000480

**Published:** 2021-09-24

**Authors:** Belen Herce-Hagiwara, Yee Mon Thu

**Affiliations:** 1 Grinnell College; 2 Allegheny College

## Abstract

Sgs1p in *Saccharomyces cerevisiae* belongs to the RecQ helicase family. Sgs1p is involved in recombination during DNA damage repair and sumoylation of Sgs1p is one mechanism by which the protein is regulated. To further understand the significance of Sgs1p sumoylation in DNA damage repair, we examined the genetic interaction between *SGS1* SUMO mutants and a mutant of *SRS2*, the protein product of which also prevents aberrant recombination structures. We observed that *SGS1-SuOff*, a mutant in which Sgs1p cannot be sumoylated, attenuates the mild sensitivity of *srs2*Δ**cells to methyl methane sulfonate.

**Figure 1:**
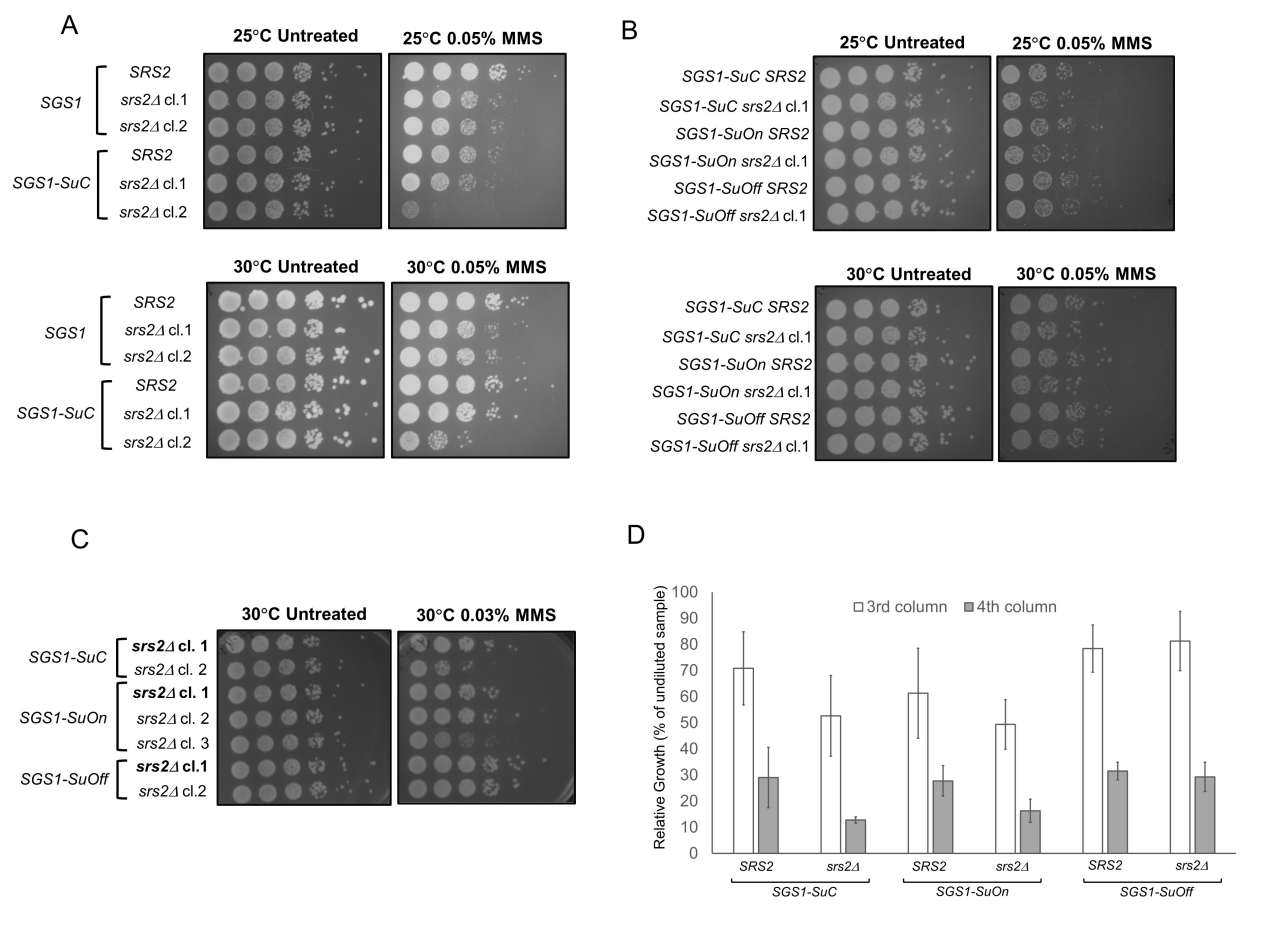
**(A)** MMS sensitivity of *SGS1*, *srs2*Δ, *SGS1-SuC* and *SGS1-SuC* *srs2*Δ strains. Cells were grown on YPD or YPD+0.05% MMS plates at indicated temperatures for 2-3 days and images were taken. **(B)** MMS sensitivity of *SGS1-SuC*, *SGS1-SuC srs2*Δ, *SGS1-SuOn*, *SGS1-SuOn srs2*Δ, *SGS1-SuOff* and *SGS1-SuOff srs2*Δ strains. Cells were grown on YPD or YPD+0.05% MMS plates at indicated temperatures for 2-3 days and images were taken. (**C)** MMS sensitivity of different clones of *srs2*Δ in *SGS1-SuC*, *SGS1-SuOn* or *SGS1-SuOff* background. Cells were grown on YPD or YPD+0.03% MMS plates at indicated temperatures for 2-3 days and images were taken. Clones indicated in bold were used for later experiments. **(D)** Quantification of the 3^rd^ and 4^th^ columns (2^nd^ and 3^rd^ dilutions) from the growth plates with MMS at 30°C. Data represent the relative growth: (growth of a specific dilution/growth of undiluted sample)x100. Quantification was derived from three independent experiments.

## Description

The RecQ helicases play a crucial role in the maintenance of genome stability. The importance of these helicases is most apparent in genetic diseases associated with RecQ helicases. In human, defects in *BLM*, *RECQL4* or *WRN* result in Bloom’s syndrome, Rothmund–Thomson syndrome and Werner’s syndrome, respectively (Ellis *et al.* 1995; Yu *et al.* 1996; Kitao *et al.* 1999). A common characteristic of all these syndromes is a predisposition to genome stability and cancer (Croteau *et al.* 2014). In *Saccharomyces cerevisiae,* Sgs1p is a RecQ helicase homolog. Sgs1p functions as part of the STR complex (Sgs1p-Top3p-Rmi1p complex) and regulates recombination to prevent illegitimate structures (Croteau *et al.* 2014). For instance, cells deficient in *SGS1* accumulated Rad51p-dependent aberrant DNA structures at replication forks when cells were treated with methylmethane sulfonate (MMS) (Liberi *et al.* 2005). How Sgs1p regulates recombination, in part, depends on sumoylation, a process in which a SUMO (small ubiquitin-like modifier) peptide is covalently conjugated to target proteins. In response to DNA damage, Sgs1p becomes sumoylated (Branzei *et al.* 2006; Bermúdez-López *et al.* 2016; Bonner *et al.*. 2016). Sumoylation of Sgs1p is mediated by an E3 SUMO ligase, Mms21p and this modification supports the ability of Sgs1p to resolve aberrant DNA structures at replication forks (Branzei *et al.* 2006; Bermúdez-López *et al.* 2016; Bonner *et al.* 2016).

Although recombination events help to overcome the impediments at replication forks, failure to regulate these processes can become the source of genome instability. Thus, it is not surprising that more than one pathway function at replication forks to prevent illegitimate recombination. Srs2p is another helicase responsible for inhibiting aberrant DNA structures in *S. cerevisiae*. Although Sgs1p and Srs2p are both 3’-to-5’ helicases and generally considered to be anti-recombinogenic, almost synthetic lethal phenotype of *sgs1 srs2* mutants implies that they function in independent pathways (Gangloff *et al.* 2000; Marini and Krejci 2010). Consistent with this idea, a recent study detailing the homologous recombination (HR) process involving Sgs1p and Srs2p uncovered that these helicases target different types of D-loops during HR (Piazza *et al.* 2019). However, genetic data suggest that Sgs1p and Srs2p have some overlapping functions. Sgs1p can suppress Srs2p deficiency in a dose-dependent manner suggesting that Sgs1p may partially compensate for the loss of Srs2p (Mankouri *et al.* 2002). Likewise, overexpression of Srs2p in *sgs1*Δreduces MMS-induced accumulation of X-molecules at replication forks (Liberi *et al.* 2005).

Based on these data, we asked if *SGS1* SUMO mutants, similar to *sgs1* knock out cells, would exhibit genetic interaction with *srs2* mutants. To this end, we genetically modified *SGS1* at the endogenous locus to create a condition in which Sgs1p cannot be sumoylated (*SGS1-SuOff*). In this strain, *SGS1* gene is fused to the domain of Ulp1p, harboring the region where SUMO contacts with Ulp1p and the isopeptidase enzymatic activity. The enzymatic activity of this domain deconjugates SUMO from the target protein, preventing Sgs1p from being sumoylated (Almedawar *et al.* 2012; Bermúdez-López *et al.* 2016; Wei and Zhao 2016). This tag indeed reduces the sumoylation of Sgs1p (Bermúdez-López *et al.* 2016). We also generated a strain to mimic constant sumoylation of Sgs1p (*SGS1-SuOn*). This strain carries a transgene encoding for Sgs1p fused to the Ulp1p isopeptidase domain that retains its affinity for SUMO but harbors a mutation that renders it catalytically dead (Almedawar *et al.* 2012; Wei and Zhao 2016). This genetic modification is known to promote sumoylation of the protein product of the target gene likely due to an increase in local concentration of SUMO (Wei and Zhao 2016). Increase in sumoylation of the target when fused to the SuOn tag has been demonstrated with Scc1p and Mcm6p (Almedawar *et al.* 2012; Wei and Zhao 2016). As a control, a similar tag with point mutations that disrupt the respective function of the SUMO-contact region and the isopeptidase activity was used (*SGS1-SuC*) (Almedawar *et al.* 2012; Wei and Zhao 2016). The sumoylation status of *SGS1-SuOn* and *SGS1-SuC* has not been examined in our study.

Wild-type *SGS1* and *SGS1-SuC* strains grew at a comparable rate and showed similar sensitivity to 0.05% MMS, suggesting that the SUMO tag does not significantly impair the growth of wild-type cells (Figure 1A). Compared to the *SGS1-SuC* strains, the SUMO mutants (*SGS1-SuOn* and *SGS1-SuOff*) did not exhibit appreciable MMS sensitivity (Figure 1B). Consistent with our data, Bermúdez-López *et al.* observed a lack of MMS sensitivity for *sgs1* mutants in which three lysine residues where Sgs1p is sumoylated are converted to arginines (Bermúdez-López *et al.* 2016). Lack of MMS sensitivity does not preclude the possibility that Sgs1p sumoylation contributes to its function. In fact, sumoylation of Sgs1p mediates its function in the context of the STR complex and discourages crossover events during double-stranded break repair (Bermúdez-López *et al.* 2016). To further understand the contribution of Sgs1p sumoylation, we took a genetic approach and studied genetic interaction between *SGS1* SUMO mutants and *srs2*. Previous studies suggest that *srs2*Δis synthetically lethal with *sgs1*Δ (Gangloff *et al.* 2000). To understand if a similar genetic interaction exists between *srs2* mutants and *SGS1* SUMO mutants, we knocked out *SRS2* in strains with wild-type *SGS1* or *SGS1* fused with different SUMO tags. *SGS1-SuC srs2*Δcl.1 exhibits MMS sensitivity similar to that of *srs2*Δclones (cl.1 and 2) (Figure 1A). However, this phenotypic similarity was not observed for *SGS1-SuC srs2*Δcl.2 and therefore, this clone was excluded from later experiments (Figure 1A). We generated independent clones of *SGS1-SuOn srs2*Δand *SGS1-SuOff srs2*Δand chose the clones that showed consistent phenotypes for downstream experiments (Figure 1C). *SGS1-SuOn srs2*Δdouble mutants exhibited similar MMS sensitivity compared to *SGS1-SuC srs2*Δcells (Figure 1B). However, *SGS1-SuOff* allele suppressed the weak MMS sensitivity that *srs2*Δ mutants exhibited (Figures 1B, C and D).

We reason that previously reported genes that exhibit a positive genetic interaction with *srs2*Δ may provide insights into the function of Sgs1p sumoylation. For example, disabling the function of Rad51p rescues the sensitivity of *srs2*Δ mutants to MMS, UV and γ-radiation, suggesting that *srs2*Δ cells accumulate Rad51p-dependent recombination structures (Aboussekhra *et al*. 1992). Srs2p can indeed disrupt Rad51p presynaptic filaments to regulate unscheduled recombination (Krejci *et al.* 2003; Veaute *et al.* 2003). Since *SGS1-SuOff* mutation also rescues the weak sensitivity of *srs2*Δ observed in this strain, one simple explanation is that deficiency to sumoylate Sgs1p prevents or attenuates unregulated Rad51p-mediated recombination in *srs2*Δ cells. The phenotype of *SGS1-SuOff srs2*Δ can be confirmed by using another SUMO mutant of Sgs1p (*sgs1-K612R* or *sgs1-3KR*). Furthermore, knocking out *RAD51* in *SGS1-SuOff srs2*Δ can also elucidate if Rad51p is responsible for the observed phenotype.

## Methods

*Generation of Yeast strains*

All strains are the derivatives of W303-1A and the relevant genotypes are shown in Table 1. *SRS2* gene was deleted by replacement of the gene with the *TRP1* auxotrophic marker amplified from pRS404 (Brachmann *et al.* 1998).

Strains with *SGS1* gene fused to the SUMO tag (*SGS1-SuC*, *SGS1-SuOn* or *SGS1-SuOff*) at the endogenous locus were generated using a two-step PCR-mediated integration (Tong and Boone 2006). Briefly, plasmids carrying the Ulp1 domain (amino acids 418-621) were acquired from the X. Zhao lab (Memorial Sloan Kettering). These tags have been previously described (Almedawar *et al.* 2012; Wei and Zhao 2016). The plasmids were used as templates to generate the first PCR product carrying the SUMO tag. The first PCR product contains 40 base pairs immediately before the stop codon of *SGS1* followed by the 5’ sequence of the SUMO tag. The first product was created using the following primers: AGGTTTTAG AAATTACCGAGGTCACTACAGAGGAAGAAAGGGTAAACCTATACCTAATCC and GTATGGTGCACTCTCAGTACAATCTCTATTTTAAAGCGTCGGTTA. The second PCR product contains the overlapping region with the first product and the *URA3* selectable marker. The second product was generated using the following primers: AGATTGTACTGAGAGTGCACCATAC and GTGTCGTAGTTATAAGTAACACTATTTATTTTTCTACTCTCTGTGCGGTATTTCACACCG. Two PCR products were purified, combined and annealed before being transformed into the intended yeast strains. Multiple clones were screened and successful integration was confirmed by PCR, followed by sequencing.

*Measuring growth and MMS sensitivity using serial dilutions*

Appropriate strains were grown overnight in YPD. From these cultures, approximately 1x 10^7^ cells were taken based on the OD_600 _readings and centrifuged. The cell pellets were resuspended in 300μl of sterile water and this sample served as the undiluted stock. Serial 10-fold dilutions were then performed in a 96-well plate using sterile water. Cells were transferred to YPD or YPD plates containing MMS using a multi-pronged spotting manifold. Strains were grown for approximately 3-4 days at indicated temperatures. Experiments were repeated three times to ensure reproducibility. Quantification of data from different experiments was performed using ImageJ. Data represent percentage of growth: (growth of a specific dilution/growth of undiluted sample)x100.

## Reagents

*Yeast Strains*

The following strains were derived from W303-1A (*MATa ura3-1 ade2-1 his3-11,-15 leu2-3,-112 can1-100 trp1-1*) acquired from the Bielinsky lab.

**Table:** 

**Strain Name**	**Genotype**	**Source**
ABy014	W303-1A	Bielinsky lab
y9	*SGS1:SGS1-SuOn (URA3)*	This study
y12	*SGS1:SGS1-SuOff (URA3)*	This study
y15	*SGS1:SGS1-SuC (URA3)*	This study
y42	*SGS1:SGS1-SuC (URA3)srs2::TRP1* cl.1	This study
y43	*SGS1:SGS1-SuC (URA3)srs2::TRP1* cl.2	This study
y44	*SGS1:SGS1-SuOn (URA3) srs2::TRP1* cl.1	This study
y45	*SGS1:SGS1-SuOn (URA3) srs2::TRP1* cl.2	This study
y46	*SGS1:SGS1-SuOn (URA3) srs2::TRP1* cl.3	This study
y47	*SGS1:SGS1-SuOff (URA3) srs2::TRP1 cl.1*	This study
y48	*SGS1:SGS1-SuOff (URA3) srs2::TRP1 cl.2*	This study
y63	*srs2::TRP1* cl.1	This study
y64	*srs2::TRP1* cl.2	This study
